# A species delimitation approach in the *Trochulus sericeus/hispidus *complex reveals two cryptic species within a sharp contact zone

**DOI:** 10.1186/1471-2148-9-171

**Published:** 2009-07-21

**Authors:** Aline Dépraz, Jacques Hausser, Markus Pfenninger

**Affiliations:** 1 Department of Ecology and Evolution, Biophore – Quartier Sorge, University of Lausanne, CH-1015 Lausanne, Switzerland; 2 Lab Centre, Biodiversity & Climate Research Centre, Biocampus Siesmayerstrasse, D-60054 Frankfurt am Main, Germany

## Abstract

**Background:**

Mitochondrial DNA sequencing increasingly results in the recognition of genetically divergent, but morphologically cryptic lineages. Species delimitation approaches that rely on multiple lines of evidence in areas of co-occurrence are particularly powerful to infer their specific status. We investigated the species boundaries of two cryptic lineages of the land snail genus *Trochulus *in a contact zone, using mitochondrial and nuclear DNA marker as well as shell morphometrics.

**Results:**

Both mitochondrial lineages have a distinct geographical distribution with a small zone of co-occurrence. In the same area, we detected two nuclear genotype clusters, each being highly significantly associated to one mitochondrial lineage. This association however had exceptions: a small number of individuals in the contact zone showed intermediate genotypes (4%) or cytonuclear disequilibrium (12%). Both mitochondrial lineage and nuclear cluster were statistically significant predictors for the shell shape indicating morphological divergence. Nevertheless, the lineage morphospaces largely overlapped (low posterior classification success rate of 69% and 78%, respectively): the two lineages are truly cryptic.

**Conclusion:**

The integrative approach using multiple lines of evidence supported the hypothesis that the investigated *Trochulus *lineages are reproductively isolated species. In the small contact area, however, the lineages hybridise to a limited extent. This detection of a hybrid zone adds an instance to the rare reported cases of hybridisation in land snails.

## Background

Mitochondrial gene sequences have become a powerful tool to identify evolutionary lineages or species in animals [[1,2], e.g. [3-5]]. Their application has often lead to the detection of divergent lineages within otherwise morphologically uniform recognised species [6]. Without any additional evidence from an explicit species delimitation approach, it is however rarely clear whether these lineages constitute reproductively isolated and/or ecologically different species or whether they are fully compatible, belonging to a single species [7,8]. The study of populations within a contact zone is thus a useful approach to assess the species status of otherwise cryptic lineages. Indeed, these areas bring genetically distinct individuals in contact and offer them the possibility to interbreed: the resulting genetic signature observed in the contact zone provides information on the degree of reproductive isolation between the lineages.

Here, we evaluated the species status of two lineages of the *Trochulus sericeus/hispidus *complex (Gastropoda: Pulmonata: Helicoidea: Hygromiidae) in an area of co-occurrence. These divergent mitochondrial lineages had been previously identified in a phylogenetic study of character evolution on the origin and the function of the hair-like shell structures in the genus [9]. While some species (i.e. *T. villosus*, *T. montanus *or *T. caelatus*) clearly matched a distinct genetic lineages each, other morphologically described species (i.e. *T. plebeius*, *T. striolatus*, *T. sericeus *and *T. hispidus*) appeared to consist of several divergent mitochondrial lineages (9 to 11% sequence divergence); the taxonomic status of the latter remained undefined due to the lack of other (e.g. morphological) criteria of discrimination [9].

This came as no surprise as the genus *Trochulus *has triggered a remarkably long taxonomic debate on species numbers and limits [10]. The large morphological variability within *Trochulus *and the multitude of transition forms led some authors to describe dozens of species [Locard in [11]] while others, based on the same shell morphological and genital anatomical evidence, recognised only a single one [11,12]. Species delimitation based on morphological features in land snails is often critical: shells have been shown in several cases to be strongly influenced by environmental conditions during growth [13,14], whereas genital anatomic criteria, whose dependence on the environment has not yet been assessed, require minute dissections and often rely, such as for *Trochulus *spp., on subtle differences in size ratios [15].

Yet, a large mitochondrial sequence divergence *per se *does not necessarily warrant species status in land snails. Unusually high levels of genetic diversity within populations have been shown for some gastropod taxa [16-18] while other studies have demonstrated the presence of "good" cryptic species [[19], e.g. [20,21]]. We used both mitochondrial and nuclear markers along with shell morphometrics to answer the following questions: (1) Is the mitochondrial divergence between the lineages reflected in the nuclear gene-pool, *i.e*. what is the degree of their reproductive isolation? (2) Are there consistent differences in shell shape among the identified lineages or are they truly cryptic? (3) Does the combination of these three datasets provide sufficient evidence to support the hypothesis of two distinct species?

## Results

### Distribution of mitochondrial lineages

**Figure 1:**
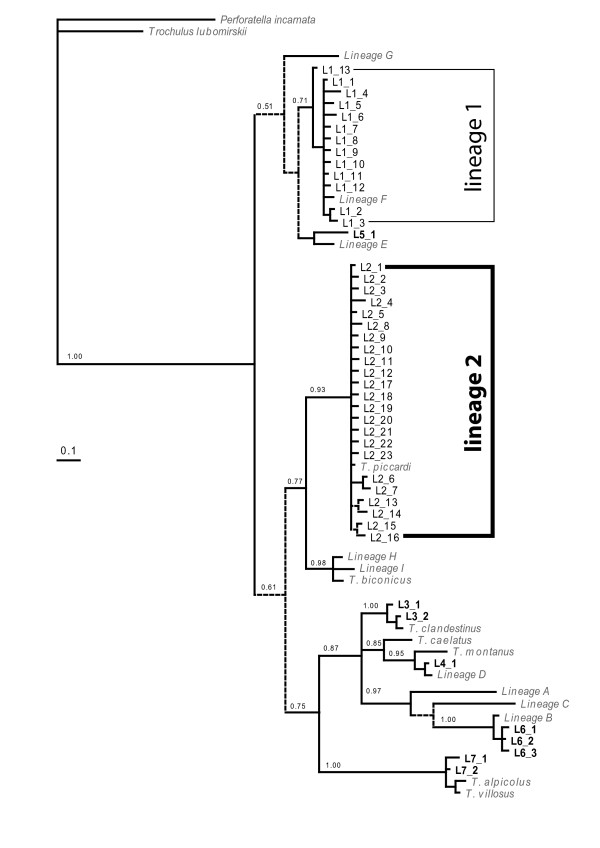
**Bayesian haplotype tree of *Trochulus *16SrRNA gene fragments**. Branches with a posterior probability lower than 50% are dashed. *Perforatella incarnata *and *Trochulus lubomirskii *were included as outgroups. The 44 haplotypes clustered into seven lineages (L1 to L7). Most of them had previously been identified (lineages A-I as in [9]).

Two hundred and twenty four individuals were sequenced for a 16SrRNA gene fragment. The mitochondrial haplotype tree clustered the sampled individuals into 47 haplotypes (GQ253517–GQ253561) grouped into seven lineages (lineages 1 to 7; Figure 1), all of which were also identified in a previous study [9]. Neighbour-joining and Maximum parsimony methods revealed the same clusters (data not shown). Genetic divergence within lineage ranged between 0.3 and 0.4% and between lineages between 5.5 – 16.7%. Lineages 1 and 2 included most of the observed haplotypes (13 and 23 haplotypes respectively) and accounted for 89% of all individuals (39% and 50% of the total sample respectively) (Table 1). Lineages 1 and 2 diverged by 7.3%. Finally, the mitochondrial tree pointed out a series of lineages (i.e. [3-7]), largely distributed among recognised *Trochulus *species: these were individuals sampled as juveniles and misidentified (Figure 1); they were ruled out from the subsequent analyses.

**Table 1:** Mitochondrial (mtDNA) and Nuclear (ncDNA) data.

				**mtDNA**				**ncDNA**	
					
**Locality**	**Coord E**	**Coord N**	**N**	**lineages **(haplotypes)	**% L1/L2**	**N. div**.	**SD**	**N_alleles_**	**SD**
**Albeuve**	7.035617	46.504849	18	**L2 (1,2)**	0/100	0.0004	± 0.0008	12.2	± 2.3
**Broc**	7.094758	46.614377	19	**L1 **(11)/**L2 **(1,16,17,20,23)	4.8/95.3	0.0004	± 0.0008	13.3	± 2.9
**Cerniat**	7.152821	46.629641	6	**L1 **(1,6,8,11)/**L4 **(1)/**L7 **(2)	77.8/0	0.0704	± 0.0392	8.0	± 1.5
**Corbières**	7.106006	46.663751	6	**L1 **(1)/**L2 **(1)/**L3 **(2)	25/50	0.0727	± 0.0412	10.7	± 3.1
**Enney**	7.091985	46.572629	14	**L1 **(1,4)/**L2 **(1,6,7,19,21,22)/**L6 **(1,3)/**L7 **(1)	11.2/66.9	0.0831	± 0.0430	15.2	± 1.5
**G. de la Jogne**	7.118248	46.606853	19	**L1 **(1,5,10,11,12)/**L2 **(1)	84.4/15.8	0.0286	± 0.0156	14.3	± 2.5
**Grandvillard**	7.061215	46.538779	15	**L2 **(1,4,9,14,15,18)	0/100	0.0086	± 0.0056	13.0	± 2.2
**La Roche**	7.121574	46.689451	9	**L1 **(1)/**L2 **(1)/**L3 **(1,2)/**L6 **(2)	53.3/6.7	0.0872	± 0.0456	13.0	± 2.5
**Le Ru**	7.121526	46.675301	9	**L1 **(1)/**L7 **(2)	84.6/0	0.0499	± 0.0270	11.3	± 4.3
**Marly**	7.144454	46.765489	14	**L1 **(1,2,3,7,9)/**L3 **(2)/**L6 **(1)	82.4/0	0.0532	± 0.0281	14.8	± 3.4
**Rossens**	7.110557	46.714706	14	**L1 **(1,2,11)/**L2 **(1)	73.4/26.7	0.0357	± 0.0195	13.8	± 2.4
**Rossinière**	7.065384	46.463697	18	**L2 **(1,2,8,10,11,12)/**L5 **(1)	0/90	0.0253	± 0.0140	14.7	± 2.4
**V. de Motélon**	7.156845	46.596845	17	**L1 **(1)/**L2 **(1,3,5,13)/**L7 **(2)	19/66.8	0.0655	± 0.0339	12.5	± 2.4
**Villarvolard**	7.106200	46.638816	14	**L1 **(1,11,13)	73.4/26.7	0.0371	± 0.0202	11.5	± 2.1

For each locality, the East and North geographical coordinates (WGS84), the haplotypes sampled, the percentage of L1 and L2 lineages (% L1/L2) and the nucleotide diversity (N. div.) with the standard deviation (SD) are given. For each sampled locality, the number of genotyped individuals (N), the average number of observed alleles over the 6 loci (N_alleles _± st. dev.; SD) are given.

**Figure 2:**
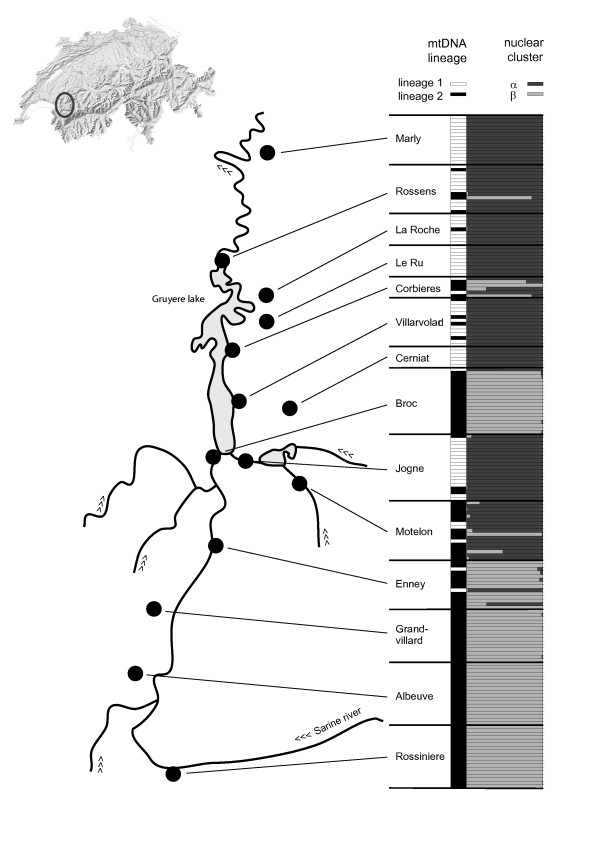
**Spatial distribution of nuclear clusters and haplotype lineages**. The spatial distribution of the sampling sites in the Sarine valley (Switzerland) is given together with the cyto-nuclear composition of the individuals sampled. Each horizontal bar represents a single individual, the first column shows the haplotype lineage (white = lineage 1, black = lineage 2) and the second column shows the estimated nuclear composition of the multilocus microsatellite genotype (dark grey = cluster α, light grey = cluster β).

Lineage 1 was predominant in the northern lower parts of the Sarine valley, while lineage 2 was preferentially found upstream in the South. There were, however, several sites in the middle of the valley where both lineages co-occurred (Figure 2).

**Figure 3:**
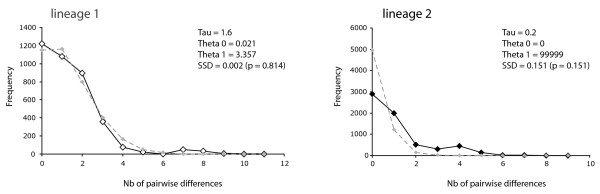
**Mismatch distributions of haplotype lineages**. The lineage 1 shows a signal of population expansion, which is not the case of lineage 2. The observed distribution is in full stroke, the simulated distribution in a dashed line. For each lineage, expansion statistics are given.

In addition, as evidenced by the mismatch distributions, lineage 1 contained a signal of spatial expansion, while lineage 2 did not (Figure 3).

### Spatial nuclear clustering and cyto-nuclear association

The spatial clustering approach with K = 2 of microsatellite multilocus genotype data from 192 individuals revealed a clear pattern: one hundred and two individuals were attributed to one cluster (α), 83 to another (β); all with posterior probabilities larger than 0.9. Seven individuals had genotypes that appeared to be of mixed origin (i.e. with posterior probabilities for each cluster between 0.1 and 0.9). These results were identical for the best 15% of runs. Geographically, cluster α was largely distributed in the north, downstream of the Sarine valley, while cluster β was preferentially found upstream in the south (Figure 2). The contribution of alleles to these clusters were visualised with a factorial correspondence analysis (see additional file 1).

There was a highly significant association of nuclear clusters with mitochondrial haplotype lineages (N = 192, p < 0.001 exact rxc contingency test, Table 2); lineage 1 being associated with nuclear cluster α and lineage 2 with cluster β. This association remained significant when only mixed populations were considered (p < 0.001). However, the association was not categorical: 22 individuals with lineage 2 had cluster α and one individual *vice versa*. Finally, all seven individuals classified as nuclear intermediates had a lineage 2 mitochondrial genome (Table 2).

**Table 2:** Cyto-nuclear association

	**cluster α**	**hybrid**	**cluster β**
**Lineage 1**	80	0	1
**Lineage 2**	22	7	82

Results of the assignments based on the nuclear genotypes into two clusters (**α and β**). Lineage 1 and 2 refer to the mitochondrial group. The rxc exact contingency table test on independence was highly significant (p < 0.001).

The sampling sites with exclusively 1/α and 2/β individuals were situated in the North and South margins of the sampling area, respectively (Figure 2). The sampling sites where both mitochondrial lineages and/or nuclear cluster co-occurred were all in the middle section of the valley (i.e. between sampling sites Enney and Rossens) and all were close to either the rivers or the reservoir lake (Figure 2).

### Shell variation

**Figure 4:**
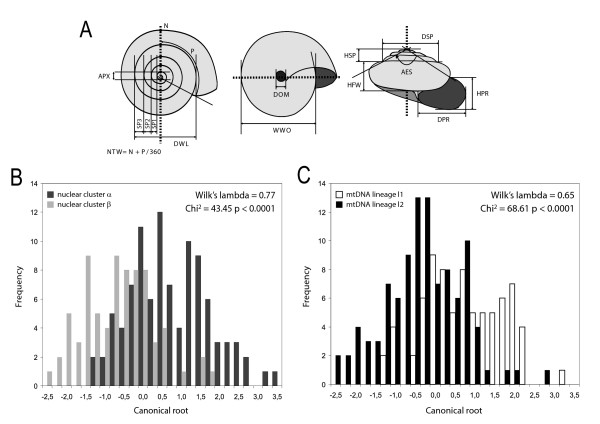
**Discriminant analyses of shell morphometric data**. A: Schematic representation of the 14 measurements taken on the shell from above, below and the front. Details on the measures are given in the text. Dotted lines represent orientation axes. B: Frequency plot of canonical scores on canonical root of discriminant analyses on morphometric variables grouped according to nuclear cluster membership (>90%). N = 171. Variables retained in the model: HFW (standardised coefficient = 0.79), HSP (0.71), DOM (0.69) and AES (0.67). Grouping resulted in 77.2% correct classifications. C: Grouped according to mitochondrial haplotype. N = 178. Variables retained in the model: HFW (0.86), HSP (0,83) and AES (0.80). Grouping resulted in 68.0% correct classifications.

A principle component analysis showed no tendency to group the individuals either according to mitochondrial lineage or nuclear cluster, and this along any of the significant axes (data not shown). Discriminant analyses, however, with grouping according to nuclear cluster or mitochondrial lineage were both significant (Figure 4). Grouping according to nuclear cluster differentiated the snails on variables such as HFW (standardised coefficient = 0.79), HSP (0.71), DOM (0.69) and AES (0.6) which tended to be slightly larger on cluster α individuals. This resulted in 78% correct posterior classifications. Grouping after mitochondrial lineage was less effective with only 69% of the cases correctly classified; the same variables were important, except DOM.

## Discussion

### Mitochondrial lineages constitute good species

The use of a short 267 bp 16SrRNA gene fragment for identification purposes has shown the power of this approach [22]. A couple of individuals morphologically similar to the *Trochulus *complex or juveniles from other species were detected due to their genetic divergence. While it is possible that these lineages interact among each other, these individuals were excluded from further analysis to ensure a rigorous statistical analysis.

The highly significant association between mitochondrial lineage (1 or 2) and nuclear cluster (α or β) in the contact zone of these parapatric lineages argues for two distinct gene-pools (Table 2) and, hence, a specific distinctness of these evolutionary lineages, satisfying the criteria of most species concepts, in particular the unified species concept [23]. Such a picture might have been also produced by very recent contact. But even considering the low dispersal capacity of land snails, the lineages could have come in contact at any time in the Holocene after the retreat of the glacier from the valley, given the small geographic scale of the study. The chances are thus minimal that the contact zone formed only within the last few generations (Figure 3). We expect therefore that free interbreeding of the lineages for many generations would have resulted in completely intermixed genotypes in the contact zone and produced a deeper zone of reciprocal introgression. The rarity of such mixed genotypes and the spatially restricted area of introgression argue for some degree of reproductive isolation. This potential reproductive isolation does not allow equating the sampling sites with freely reproducing populations, which precludes their model dependent population genetic analysis.

If one applies the fastest mitochondrial divergence rate of 5% per million years ever suggested for land snails [16], a sequence divergence of more than 7% between the lineages suggests that they split at least 1.4 million years ago. Slower, more realistic rates would entail an even older divergence. This implies that the lineages were separated for at least most of the Pleistocene, giving them ample time to diverge and speciate.

This distinctness is also reflected in the shell morphology. Both mitochondrial and nuclear data appeared to be suitable statistical predictors of different morphological groups. The nuclear cluster performs slightly better, which we attribute to the few cases of mitochondrial introgression of lineage l2 haplotypes into nuclear background α, blurring the picture (see below). One could attribute the overlap between the two morphogroups to extensive mutual introgression at the relevant genes. This is however unlikely: even the populations furthest apart from each other (and thus least prone to extensive exchange) have overlapping morphologies (data not shown). Moreover, the possibility to statistically tell the two species apart and the approximately normally distributed factor scores (Figure 4B, 4C) suggest that at least some of the quantitative shell shape differences are governed by multiple divergently evolved genes [24]. The observed morphological shell differences are thus rather due to drift than different biological function.

However, a classification success rate of only 78% and 69% respectively, means that it is impossible to reliably differentiate the two species' shells based on extensive morphometric measurements (Figure 4), let alone to distinguish them visually. In practice, this means that the two species will remain cryptic unless molecular markers are used for identification. While the lineage 1/cluster α is yet unnamed, 2/β individuals may be attributed to *Trochulus piccardi *[25]. Indeed, the lineage 1 individuals, in addition of grouping with the type individual of that species on the mitochondrial phylogeny, have been sampled within the geographical range described for this species [25].

The distribution of *T. piccardi *that appears to be restricted to the upper Sarine valley and surrounding regions suggests *in situ *evolution [25]. Such a hypothesis requires the continuous presence of suitable habitat throughout the Pleistocene glaciation cycles, probably scattered over steep south-facing slopes. However, such refugia need not to be large for such poor dispersing, small animals: it has been shown that they can survive on very small areas, provided they are surrounded by other population patches [26].

The current distribution of the other *Trochulus *species (*i.e*. the lineage 1) lacks preciseness as this study's sampling scheme did not reach its northern limit. It can nevertheless be argued that it probably evolved in its own, not yet located Pleistocene refugium and got into secondary contact with *T. piccardi *in the course of a Holocene range expansion (Figure 3).

A pattern of speciation in isolation may turn out to be quite common within *Trochulus*: several species with very restricted ranges in mountainous regions have been described such as *T. graminicola *(FALKNER 1973), *T. montanus *(STUDER 1820), *T. caelatus *(STUDER 1820) or *T. biconicus *(EDER 1917). Generally speaking, it has been repeatedly put forward that alpine areas could provide pocket refugia for gastropods [27-30]. Even though a Pleistocene survival of land snails in alpine or other Northern refugia has not been traditionally considered [31], accumulating evidence suggests their importance for gastropod biodiversity [32-35].

### Mitochondrial introgression in a hybrid zone

A few individuals with apparently admixed nuclear genotypes as well as some with the "wrong" mitochondrial genome could be detected (Figure 2). These intermediates suggest that, despite the relatively large evolutionary distance among the species, occasional hybridisation can take place (Figure 1, [9]).

Interestingly, the introgression of mitochondrial genomes seems to be directional: only a single lineage 1/cluster β individual was found which is little as compared to the 22 lineage l2/cluster α individuals; besides the admixed nuclear genotypes all carried the lineage 2. Even though the sample sizes of the admixed populations were too small to perform formal tests on cytonuclear disequilibrium [36], the apparent directional introgression of lineage 2 mitochondrial haplotypes into cluster α nuclear background begets the question whether this is due to intrinsic reproductive barriers and/or unidirectional gene-flow downstream the Sarine river. Differential mating preference, the usual explanation for the observed pattern, is unlikely in the present case: since these snails are reciprocally mating hermaphrodites (i.e. they both receive the sperm of the other individual to fertilise their eggs), the nuclear and mitochondrial genomes of both individuals are expected to be transmitted. Given the low dispersal capacity of land snails and their more or less isolated populations [37-40], it is anyway likely that we are dealing here with a mixed hybrid zone [36], where isolated hybrid populations may take different evolutionary trajectories. A hint in this direction was the somewhat peripheral sampling site Motelon (Figure 2), where nuclear cluster α is associated to lineage 2, contrary to all other sites: it may be due to one or a few introgression events, followed by drift in isolation.

## Conclusion

The application of several lines of evidence allowed rejecting the hypothesis of a single species consisting of divergent lineages, despite occasional hybridisation in a contact zone. The detection of a putative hybrid zone in *Trochulus *adds to the surprisingly low number of cases of hybridisation in land snails [41-44], despite the ubiquitous presence of hybridisation in animal taxa [45]. This system – two cryptic hermaphrodite species with a small hybrid zone – seems excellently suited for further investigations on the evolutionary forces shaping species diversity in mountainous habitat rich regions, with a focus on the evolutionary consequences of hybridisation.

## Methods

### Sampling

Fourteen sampling sites in a previously identified area of co-occurrence of mitochondrial lineage F and *Trochulus *nov. spec. as defined in Pfenninger *et al*. 2005 (subsequently described as *T. piccardi *PFENNINGER & PFENNINGER[25] were sampled in the Swiss Western Prealps, along the Sarine valley (Figure 2). As the attribution of *Trochulus *individuals to a certain species based on shell morphology alone is difficult, all apparently adult *Trochulus sericeus/hispidus *individuals found were sampled. The collected snails were brought alive to the lab and frozen at -80°C. The individuals were then pulled out of their shell and stored at -80°C for genetic analyses; the shell was kept for morphometric measurements.

### Sequencing and mitochondrial analyses

Total DNA was extracted from a piece of foot muscle following a modified salt/chloroform procedure by adding one step of chloroform-isoamylalcohol (24:1) [46]. The mitochondrial haplotypes were determined by sequencing a 267 bp fragment of the 16SrRNA gene using universal primers [47]. Reactions were carried out in a 50 μl volume containing 2 μl of template, 1.5 units of Taq DNA Polymerase (Qiagen), 1× of its buffer, 1000 μM of MgCl_2 _and 100 μM of dNTP and 0.4 μM of each primer. The PCR was run on a DNA Thermal Cycler (Perkin Elmer, Norwalk, CT) starting with an initial denaturation at 94°C for 3 minutes, followed by 10 cycles at 44°C annealing temperature (50 sec at 92°C, 30 sec at 44°C and 40 sec at 72°C) and 35 cycles at 48°C annealing temperature (30 sec at 92°C, 30 sec at 48°C and 40 sec at 72°C), and ended by a 3 minute final extension at 72°C. After amplification, all samples were controlled on a 1% agarose minigel stained with ethidium bromide and run in a 1× TBE buffer. The PCR products were then purified with a QIAQuick PCR Purification Kit (Qiagen) according to the manufacturer's instructions. The purified DNA was eluted in 30 μl of dH_2_O. Samples were sent to a sequencing company (Microsynth AG, Balgach, Switzerland). Sequences were edited, aligned on SEQUENCHER 3.0 (Gene Codes Corporation) and the alignment manually corrected using SEAVIEW[48].

We constructed a haplotype phylogeny to visualise haplotype clusters using MRBAYES v. 3.1 [49]. A GeneBank reference sequence for each of the nine previously identified lineages was added [Lineages A to I as in [9]], as well as sequences of *Trochulus biconicus*, *T. caelatus*, *T. clandestinus*, *T. alpicolus*, *T. piccardi *and *T. lubomirskii*. *Perforatella incarnata *was included as the outgroup species. We ran MODELTEST V.3.7[50] on this extended dataset and selected a GTR+R+I model, assuming a gamma-shaped rate variation and invariant sites. We ran two runs of four Metropolis coupled Monte Carlo Markov chains (MC^3^) for 10 million generations, sampling every 500^th ^generation and discarding the first 100'000 trees as burn-in. Convergence was monitored by checking that the average standard deviation of the split frequencies (i.e. the differences in likelihood between the 2 runs) were below 0.1% (MRBAYES).

Episodes of population growth and decline may also leave characteristic signatures in the distribution of pairwise nucleotide differences of populations (i.e. mismatch distribution). The mismatch distributions under a sudden expansion model were computed for the two mitochondrial lineages (L1 and L2) with ARLEQUIN v.3.1 [51]. This model assumes that an initial population at equilibrium with θ = θ_0 _grows rapidly to a new size with θ = θ_1_, τ units of mutational time ago, where θ = *N*_*e*_*u *and τ = 2 *ut *(*N*_*e *_= effective population size, *u *= mutation rate and *t *= time since the expansion in generations). Goodness-of-fit tests [52] of the observed to the expected distribution were computed. The confidence intervals for τ were obtained from 1000 bootstrap replicates.

### Genotyping and spatial genotype clustering

The individuals belonging to the two mitochondrial lineages in focus were scored at 6 microsatellite loci – TROA6, TROA111, TROB5, TROB108, TROB111 and TROB112 – following the published PCR conditions [53]. Amplifications were run on an ABI 3100 capillary sequencer (Applied Biosystems) and scored with GENEMAPPER v.3 (Applied Biosystems).

We applied a Bayesian spatial population genetics clustering algorithm implemented in TESS 1.2 to obtain individual posterior membership probabilities of nuclear gene-pools [54,55]. The method is based on a hierarchical mixture model where the prior distribution of cluster labels is defined as a Hidden Markov Random Field (HMRF) on a spatial individual tessellation network. The program seeks genetic structure from individual multilocus genotypes sampled at different geographical locations without assuming predefined clusters. It returns the membership probabilities and cluster assignments of the individuals. In the initial field sampling, we did not record individual sampling points, as it is required by TESS. Therefore, we assigned a randomised sampling point to each individual, drawn from a square of 100 × 100 m around the recorded sampling location as given in Table 1. This proceeding actually models very well the sampling of land snails.

As the sampling sites are located along a river valley without *a priori *obstacle to dispersal, we left the neighbourhood diagram unmodified. We used the admixture model in a Monte-Carlo-Markov-Chain approach for the microsatellite data set, the interaction parameter set to 0.6 and the allele frequency model parameter to 1.0. We were not interested in intraspecific population structure but in gene pool membership; the maximum number of clusters was therefore set to two. We performed 200 runs with 50,000 sweeps per run with a burn-in of 12,000 sweeps, which was sufficient to reach stationarity. We used the program CLUMPP[56] to obtain averages of the 15% runs with the highest likelihoods. We performed a G-test to test for independence of mitochondrial lineage from nuclear cluster.

### Shell morphometrics and discriminant analysis

A hundred and seventy eight adult individuals were selected for shell morphometric analyses according to three criteria: the individual (i) belonged to one of the two major mtDNA lineages and microsatellite data was available, (ii) had a shell with at least five complete whorls and (iii) had a shell undamaged as much as to allow all measures. Fourteen measurements were taken on calibrated digital pictures of the shells (Figure 4). From above, we measured the decimal number of whorls (NTW computed as the number of complete whorls plus the partial whorl (in degrees; divided by 360), the width of the apex (APX), the width of the first (SP1), second (SP2) and third whorl (SP3) and the diameter of the spire without the last whorl (DWL). From underneath, the diameter of the umbilicus (DOM) and the width of the shell without the opening (WWO) were taken. Finally, from a side perspective, we measured the height of the spire (HSP), the height of the first whorl (HFW), the diameter of the peristoma (DPR), the height of the peristoma (HPR), the diameter of the spire (DSP) and the shape, measured as the angle formed by the apex and the most external sutures (AES) (Figure 4). As first exploratory approach, a principle component analysis was performed. Two discriminant analyses were run on the morphometric variables: a first with nuclear cluster as grouping factor, a second with the mtDNA lineage. All analyses of the morphometric data were performed with STATISTICA 7 [57].

## Authors' contributions

AD designed the study, sampled the snails, performed the lab work and drafted the manuscript. JH took part in the design, performed the morphometric measurements and drafted the manuscript. MP analysed the data and drafted the manuscript. All authors read and approved the final manuscript.

## Supplementary Material

Additional file 1 Factorial correspondence analysis of microsatellite data. Click here for file
