# Supplementary material for: A species delimitation approach in the Trochulus sericeus/hispidus complex reveals two cryptic species within a sharp contact zone
Source: BMC Evol Biol. 2009 Jul 21;9:171. doi: 10.1186/1471-2148-9-171 (PMC2724411; doi:10.1186/1471-2148-9-171)
Supplement: Additional file 1 — Factorial correspondence analysis of microsatellite data. [file 1471-2148-9-171-S1.pdf]

Additonal material file1

Factorial correspondence analysis (FCA) was applied on multilocus genotypes to explore the distribution of genetic variation graphically (GENETIX 4.04 software).

Belkhir K, Borsa P, Chikli L, Raufaste N, Bonhomme F (1996) GENETIX 4.04, Logiciel Sous Windows TM Pour la Génétique des Populations. Montpellier: Laboratoire Génome, Université Montpellier.

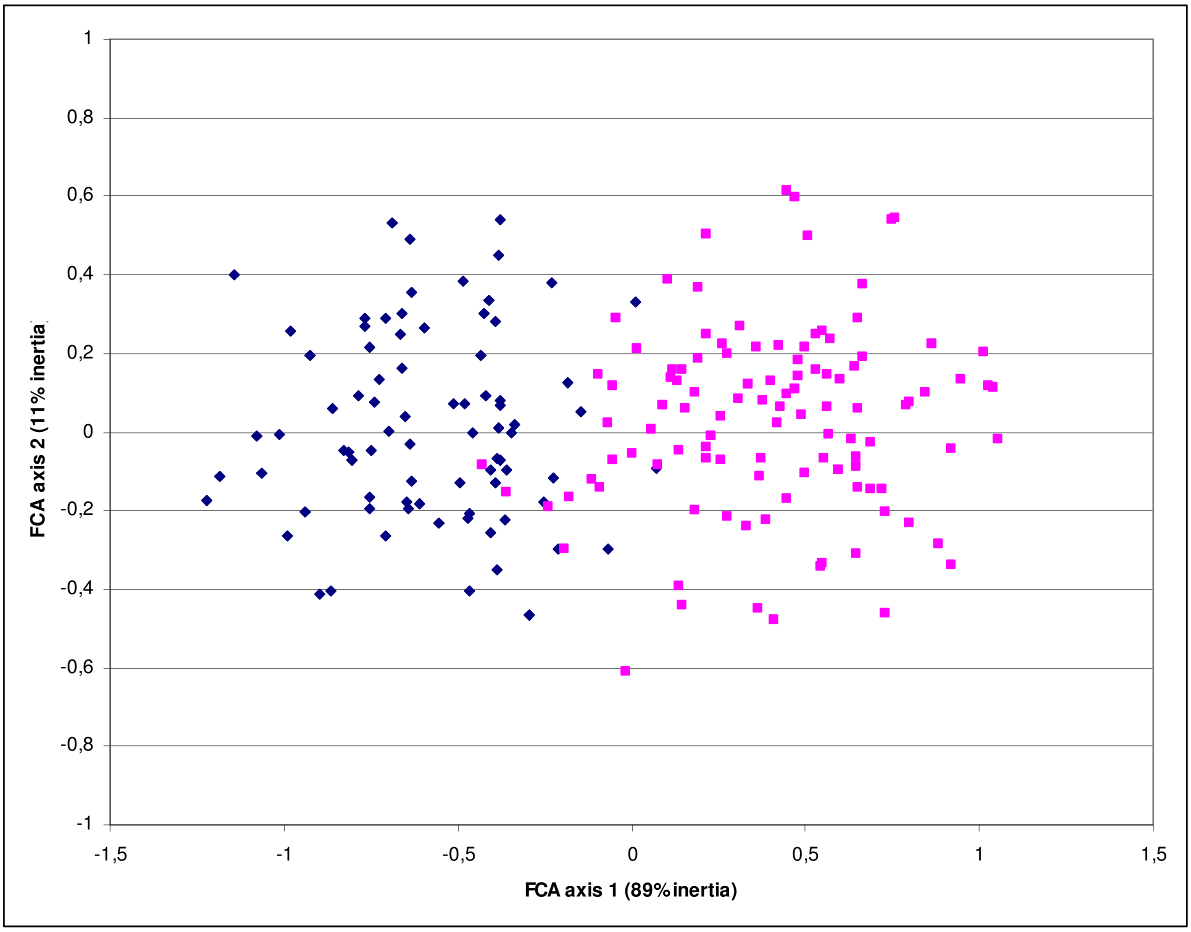

Figure 1: Factorial Correspondence Analysis of nuclear microsatellite variation. Shown is the position of the individuals, marked by their haplotype group, on the first two axes. Axis 1, representing 89% of total inertia, separates the haplotype groups with little overlap.

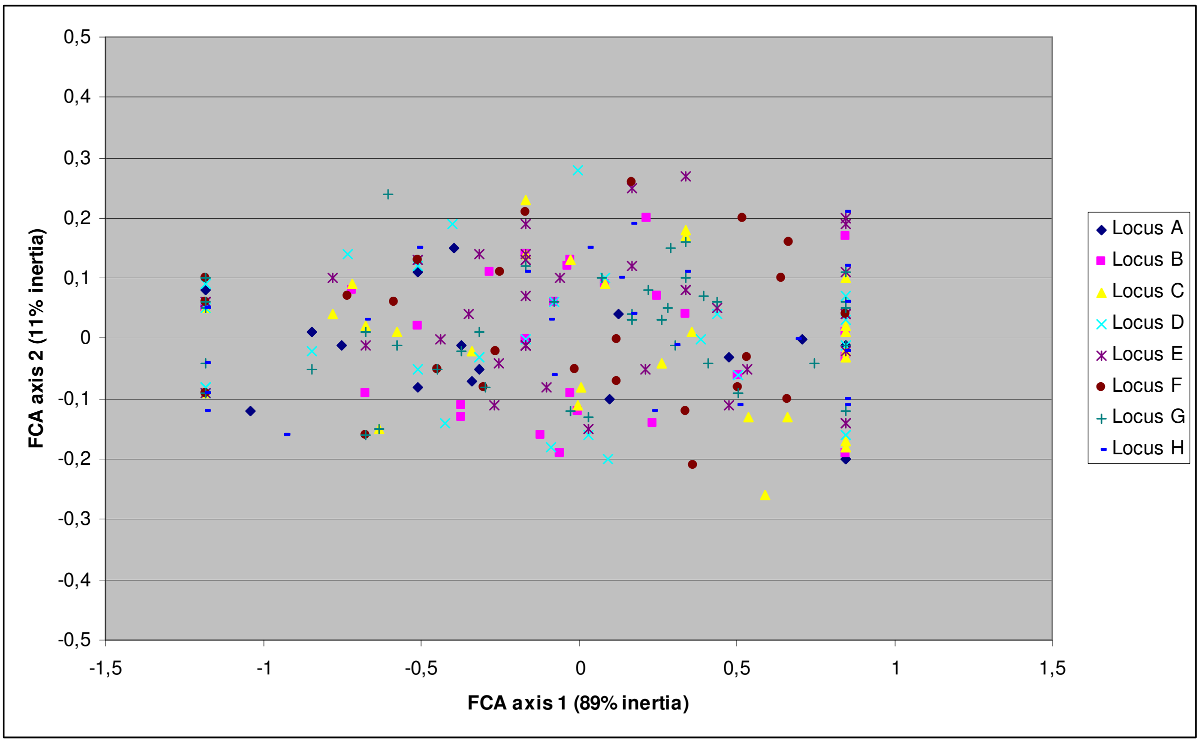

Figure 2. Factorial Correspondence Analysis of nuclear microsatellite variation. Shown is the position of the alleles, grouped per locus, on the first two axes. Alleles with the highest, respectively lowest score on the first axis are private alleles of the lineages. Note, however, that alleles seemingly shared among lineages need not necessarily indicate common ancestry as the length of microsatellites may represent homoplasious character states.
